# Intensified cytarabine dose during consolidation in adult AML patients under 65 years is not associated with survival benefit: real-world data from the German SAL-AML registry

**DOI:** 10.1007/s00432-022-04356-9

**Published:** 2022-09-28

**Authors:** Maher Hanoun, Leo Ruhnke, Michael Kramer, Christine Hanoun, Kerstin Schäfer-Eckart, Björn Steffen, Tim Sauer, Stefan W. Krause, Christoph Schliemann, Jan-Henrik Mikesch, Martin Kaufmann, Mathias Hänel, Edgar Jost, Tim H. Brümmendorf, Lars Fransecky, Sabrina Kraus, Hermann Einsele, Dirk Niemann, Andreas Neubauer, Johannes Kullmer, Ruth Seggewiss-Bernhard, Martin Görner, Gerhard Held, Ulrich Kaiser, Sebastian Scholl, Andreas Hochhaus, H. Christian Reinhardt, Uwe Platzbecker, Claudia D. Baldus, Carsten Müller-Tidow, Martin Bornhäuser, Hubert Serve, Christoph Röllig, Study Alliance Leukemia (SAL)

**Affiliations:** 1 Department of Hematology and Stem Cell Transplantation, University Hospital Essen, Essen, Germany; 2 Department of Internal Medicine I, University Hospital Dresden, TU Dresden, Dresden, Germany; 3 Department of Internal Medicine 5, Hospital Nuernberg, Nuernberg, Germany; 4 Department of Hematology and Oncology, University Hospital Frankfurt am Main, Frankfurt am Main, Germany; 5 Department of Hematology, Oncology and Rheumatology, University Hospital Heidelberg, Heidelberg, Germany; 6 Department of Hematology and Medical Oncology, University Hospital Erlangen, Erlangen, Germany; 7 Department of Medicine A, University Hospital Münster, Münster, Germany; 8 Department of Hematology, Oncology and Palliative Medicine, Robert-Bosch-Hospital, Stuttgart, Germany; 9 Department of Internal Medicine III, Chemnitz Hospital, Chemnitz, Germany; 10 Department of Internal Medicine IV, University Hospital RWTH, Aachen, Germany; 11 Department of Internal Medicine II, University Hospital Schleswig-Holstein, Kiel, Germany; 12 Department of Internal Medicine II, University Hospital Würzburg, Würzburg, Germany; 13 Department of Internal Medicine, Hematology and Oncology, Gemeinschaftsklinikum Mittelrhein, Koblenz, Germany; 14 Department of Hematology, Oncology and Immunology, University Hospital Marburg, Marburg, Germany; 15 Department of Internal Medicine II, IAKO Bremen, Bremen, Germany; 16 Department of Internal Medicine V, Sozialstiftung Bamberg, Bamberg, Germany; 17 Department of Hematology, Oncology and Palliative Medicine, Klinikum Bielefeld Mitte, Bielefeld, Germany; 18 Department of Hematology, Oncology, Clinical Immunology, Rheumatology, Medical School, University of Saarland, Homburg, Germany; 19 Department of Hematology and Oncology, St. Bernward Hospital, Hildesheim, Germany; 20 Klinik für Innere Medizin II, Universitätsklinikum Jena, Jena, Germany; 21 Department for Internal Medicine I, University Hospital Leipzig, Leipzig, Germany; 22 National Center for Tumor Disease Dresden (NCT/UCC), Dresden, Germany

**Keywords:** Acute myeloid leukemia, Consolidation therapy, Cytarabine dosage, Real-world data

## Abstract

**Purpose:**

Higher doses of cytarabine appear to improve long-term outcome in acute myeloid leukemia (AML), in particular for younger patients. To this end, the optimal dosage of single-agent cytarabine in consolidation therapy remains elusive. Here, we assessed the impact of different dosages of cytarabine consolidation after 7 + 3 induction on outcome in a large real-world data set from the German Study Alliance Leukemia-Acute Myeloid Leukemia (SAL-AML) registry.

**Methods:**

Patients between 18 and 64 years of age, registered between April 2005 and September 2020, who attained complete remission after intensive induction and received at least one consolidation cycle with intermediate (IDAC) or high-dose cytarabine (HiDAC) were selected. To account for differences in patient and disease characteristics between both groups, the average treatment effect was estimated by propensity score weighting.

**Results:**

Six-hundred-forty-two patients received HiDAC consolidation with median dosage of 17.6 (IQR (interquartile range), 16.5–18.0) g/m^2^ for a median number of 3 cycles (IQR, 2–3), whereas 178 patients received IDAC consolidation with 5.9 (IQR, 5.7–8.6) g/m^2^ for a median of 2 cycles (IQR, 1–3). Both groups differed significantly in some important characteristics (age, sex, cytogenetic risk group, ECOG performance status, disease status, HCT-CI, number of induction cycles). After propensity score weighting for differences in patient and disease characteristics, relapse-free survival after 2 years was comparable between HiDAC-treated (55.3%) and IDAC-treated (55.6%) patients (HR = 0.935, *p* = 0.69). Moreover, no significant differences in overall survival were observed after 2 years (84.7 vs. 80.6%, HR = 1.101, *p* = 0.65). Notably, more patients treated with IDAC received allogeneic hematopoietic cell transplantation in first remission (37.6 vs. 19.8%, *p* < 0.001). Censoring for allogeneic hematopoietic cell transplantation in first remission revealed no significant survival difference with regard to cytarabine dosage. Considering only of European LeukemiaNet (ELN) favorable-risk AML patients, there was no significant difference in outcome. Of note, significantly more patients treated with HiDAC suffered from ≥ 3 CTCAE infectious complications (56.7 [95%-CI 52.8–60.6%] vs. 44.1% [95%-CI 36.6–51.7%]; *p* = 0,004). The rate of other ≥ 3 CTCAE non-hematological toxicities and secondary malignancies was comparable in both treatment groups.

**Conclusions:**

This retrospective analysis suggests no significant benefit of high-dose cytarabine compared to intermediate dosages in consolidation for AML patients under 65 years of age, independent of ELN risk group.

**Trial registration:**

NCT03188874.

**Supplementary Information:**

The online version contains supplementary material available at 10.1007/s00432-022-04356-9.

## Background

Acute myeloid leukemia (AML) is an aggressive disease, which requires intensive treatment strategies to achieve curation. The mainstay of intensive anti-leukemic therapy comprises an induction with anthracyclines/mitoxantrone and cytarabine, commonly applied as the so-called 7 + 3 regimen with seven days cytarabine and three days of daunorubicin. Depending on genetic risk, induction therapy can vary by formulation of chemotherapy or addition of targeted therapies. However, AML is characterized by a high relapse rate, indicating insufficient clearance of leukemia-initiating cells (Dohner et al. 2017). Therefore, effective post-remission strategies are urgently needed to reduce risk of relapse. For genetically defined intermediate and adverse risk patients, according ELN 2017 classification, allogeneic hematopoietic cell transplantation is the most effective consolidation. However, patient-, donor- or transplantation-related issues limit its use. In these cases, as well as for genetically favorable AML patients with a lower relapse risk, consolidating chemotherapy should be applied. Of note, there is no clear benefit of intensified post-remission chemotherapy, including intermediate or high doses of cytarabine for elderly patients, in particular for adverse risk patients (Dohner et al. 2017; Itzykson et al. 2011). For younger patients, single-agent cytarabine at high doses as consolidating treatment proved to result in similar outcome compared to multiagent chemotherapeutic protocols (Dohner et al. 2017; Miyawaki et al. 2011; Schaich et al. 2013; Thomas et al. 2011). This entails the question to define the optimal dose of cytarabine after 7 + 3 induction therapy. Previous results from the Cancer and Leukemia Group B (CALBG) study group have demonstrated an advantage for high-dose cytarabine with six applications at 3000 mg/m^2^ compared to conventional cytarabine doses of 100 or 400 mg/m^2^ for patients below 60 years of age (Mayer et al. 1994). However, there is lacking evidence for increasing cytarabine doses above 2000 mg/m^2^ compared to intermediate doses of 1000 mg/m^2^ for consolidation treatment after 7 + 3 induction therapy. Numerous trials included comparisons of high-to-intermediate doses in consolidation, which do not show any advantage to raise the cytarabine dose above 1000 mg/m^2^ twice daily (Lowenberg 2013). However, these studies often contained different induction protocols, partly including higher doses of cytarabine already during induction treatment or multiagent protocols in consolidation (Miyawaki et al. 2011; Schaich et al. 2011). The different drug combinations during induction therapy and post-remission therapy might influence the therapeutic impact of the different cytarabine consolidation schedules in variable manners. Here, we retrospectively tested the significance of high-dose versus intermediate-dose cytarabine as monotherapy after uniform 7 + 3 induction treatment in patients under 65 years of age in a large real-world data set from the German Study Alliance Leukemia-Acute Myeloid Leukemia (SAL-AML) registry.

## Methods

**Fig. 1:**
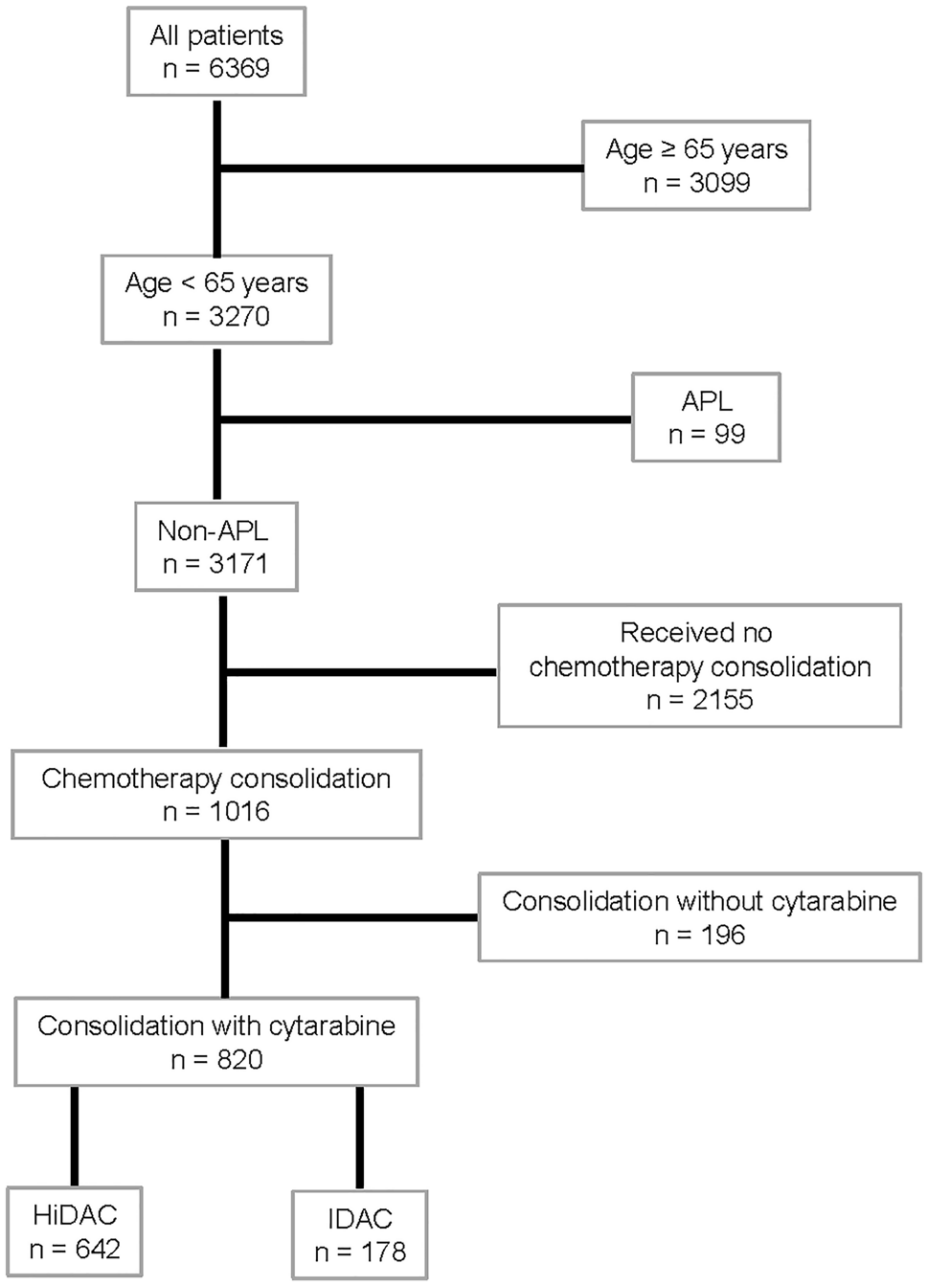
Patient selection for the present analysis. *APL* acute promyelocytic leukemia; *HiDAC/IDAC* high-dose/intermediate-dose cytarabine

Patients between 18 and 64 years of age, registered between April 2005 and September 2020 with non-acute promyelocytic leukemia, who attained complete remission after intensive induction and received at least one consolidation cycle with intermediate (IDAC) or high-dose cytarabine (HiDAC), defined as 1–1.5 g/m^2^ and ≥ 2 g/m^2^, respectively, were selected from the SAL-AML registry (Fig. 1). Patients with initially palliative treatment but subsequent complete remission were excluded. Median follow-up time was 41.4 (IQR, 18.3–65.0) months. The study protocol has been approved by the ethics committees of all participating centers and the study is registered (NCT03188874).

Overall survival (OS) was defined as time from diagnosis to death from any cause. If no death was observed, OS time was censored on date of last follow-up. Relapse-free survival (RFS) was defined as time from date of first complete remission until relapse or death from any cause, whichever occurred first. If no relapse or death was observed, RFS time was censored on date of last follow-up. To assess the impact of allogeneic hematopoietic cell transplantation in sensitivity analyses, we also calculated OS and RFS with censoring on the date of allogeneic hematopoietic cell transplantation, if this occurred before the first event of interest. For estimation of adjusted survival of IDAC- and HiDAC-treated patients according to Kaplan–Meier propensity score weights for the average treatment effect were estimated, because both groups differed significantly in some important characteristics (age, sex, cytogenetic risk group, ECOG performance status, disease status, HCT-CI, number of induction cycles). A sufficient balance was reached with these propensity score weights. To assess the differential impact of IDAC versus HiDAC, multivariable Cox regression models with interactions of IDAC/HiDAC and the variable of interest were fitted. These models were adjusted for the parameters also used for estimation of the propensity score weights. Missing values in variables were imputed with simple imputation methods, if they were used for calculation of the propensity score or for adjustment of the multiple regression models. Missing values in categorical variables were imputed with the most frequent category of the observed values, missing values in continuous variables were imputed with the median of the observed values.

## Results

### Patient disposition

Eight-hundred-twenty patients from the database of the SAL-AML registry fulfilled the criteria and were included in the analyses (Fig. 1). 178 patients were treated with approximately 6 g/m^2^ cytarabine per cycle (median 5.9 (IQR, 5.7–8.6) g/m^2^), corresponding to 6 applications of 1 g/m^2^ cytarabine, compared to 642 patients who received approximately 18 g/m^2^ cytarabine per cycle (median 17.6 (IQR, 16.5–18.0) g/m^2^), which corresponds to 6 applications at 3 g/m^2^ (Table 1). Only 2.8% or 1.6% received additional agents during consolidation. Thus, the selected cohort was almost exclusively treated with single-agent cytarabine for consolidation. IDAC-treated patients were older (median (IQR) 58.5 (49–62) vs. 50 (41–56) years, p < 0.001) and had significantly more often secondary and therapy-related AML, as well as more adverse and less favorable genetic risk features according to the ELN 2017 classification (Table 1). Of note, 116 core binding factor AML (CBF AML) were treated with HiDAC, while only 20 received IDAC for consolidation. Furthermore, IDAC-treated patients had more comorbidities according to HCT-CI score (HCT-CI ≥ 2 43.8 vs. 22.3%, *p* < 0.001). Eighty, respectively, 90% of patients have been induced with the 7 + 3 regimen. Based on German recommendations, the number of induction cycles differed significantly with more patients within the HiDAC cohort receiving 2 cycles (76.8 vs. 61.2%, *p* < 0.001). Likewise, the median number of consolidation cycles was different with two (IQR, 1–3) in the IDAC group and three (IQR, 2–3) among HiDAC-treated patients. As a result of more unfavorable risk patients in elderly patients > 60 years, significantly more patients treated with IDAC received allogeneic hematopoietic cell transplantation in first remission (37.6 vs. 19.8%, *p* < 0.001). Whereas the rate of transplantation after relapse was higher among HiDAC-treated patients (30.8 vs. 20.2%, *p* = 0.007).

**Table 1:** Patient and treatment characteristics of all patients

	IDAC (*n* = 178)	HiDAC (*n* = 642)	*p* value	*p* value (after PS weighting)
Age at initial diagnosis (years, median (IQR))	58.5 years (IQR, 49–62)	50.0 years (IQR, 41–56)	< 0.001	0.246
Female sex, no./no. Available (%)	88/178 (49.4%)	314/642 (48.9%)	0.968	0.867
AML type, no./no. available (%)				
de novo AML	147/178 (82.6%)	594/640 (92.8%)	< 0.001	0.852
sAML	9/178 (5.1)	20/640 (3.1)		
tAML	22/178 (12.4)	26/640 (4.1)		
ELN-Risk 2017 group, no./no. available (%)				
Favorable	67/165 (40.6)	336/600 (56)	< 0.001	0.915
Intermediate	74/165 (44.8)	225/600 (37.5)		
Adverse	24/165 (14.5)	39/600 (6.5)		
Core binding factor AML	20/168 (11.9)	116/604 (19.2)	0.037	0.585
Complex karyotype, no./no. available (%)	15/168 (8.9)	29/609 (4.8)	0.060	0.182
FLT3-ITD	38/161 (23.6)	123/595 (20.7)	0.486	0.927
NPM1 mut	68/168 (40.5)	280/612 (45.8)	0.258	0.663
HCT-CI				
0–1	100/178 (56.2)	498/641 (77.7)	< 0.001	0.595
2–4	78/178 (43.8)	143/641 (22.3)		
Induction therapy				
1 Cycle 7 + 3	67/178 (37.6)	151/642 (23.5)	< 0.001	< 0.001
2 Cycles 7 + 3	75/178 (42.1)	428/642 (66.7)		
7 + 3/HAM	8/178 (4.5)	33/642 (5.1)		
Others	28/178 (15.7)	30/642 (4.7)		
Number of consolidation cycles (median (IQR))	2 (IQR,1–3)	3 (IQR,2–3)	< 0.001	< 0.001
Cytarabine dose per chemo-consolidation cycle (median (IQR))	5891.85 mg/m^2^ per cycle	17,580.38 mg/m^2^ per cycle	< 0.001	< 0.001
Additional substances	5/178 (2.8)	10/642 (1.6)	0.432	0.107
Allogeneic HCT in CR1	67/178 (37.6)	127/642 (19.8)	< 0.001	< 0.001
Allogeneic HCT salvage	36/178 (20.2)	198/642 (30.8)	0.007	0.017

*HiDAC/IDAC* high-dose/intermediate-dose cytarabine; *IQR* interquartile range; *sAML* secondary AML; *tAML* treatment-related AML; *HCT-CI* hematopoietic cell transplantation-comorbidity index; *7 + 3* induction treatment with standard-dose cytarabine for 7 d and daunorubicin for 3 d; *HAM* high-dose cytarabine plus mitoxantrone; *HCT* hematopoietic cell transplantation; *CR1* first complete remission; *PS* propensity score

### Effect of cytarabine dose on outcome

**Fig. 2:**
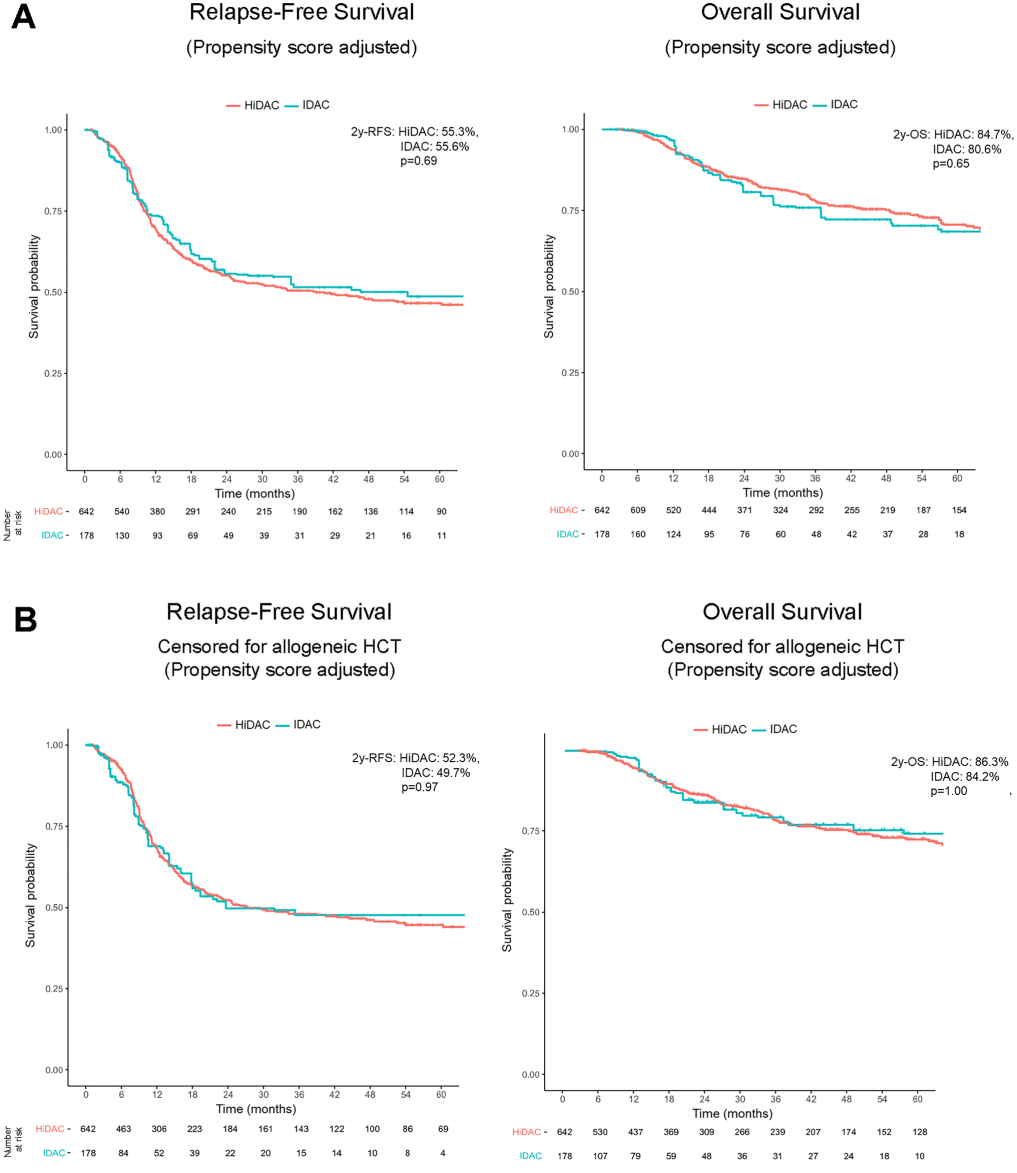
Kaplan–Meier estimates of relapse-free survival (RFS, left) and overall survival (OS, right) for all patients after propensity score adjustment (**A**) and censored for allogeneic hematopoietic cell transplantation (HCT) in first complete remission (**B**)

To explore the impact of cytarabine dose in post-remission therapy after intensive induction treatment on survival, propensity score weights were estimated for the average treatment effects, which allowed adjusting for imbalances in prognostic variables and estimating adjusted Kaplan–Meier curves. There was no difference in RFS with a 2-year survival probability of 55.3% in HiDAC- versus 55.6% in IDAC-treated patients (HR = 0.935, *p* = 0.69) (Fig. 2A). Also for OS, there were no significant differences with a 2-year OS probability of 84.7% in HiDAC vs. 80.6% in IDAC group (HR = 1.101, *p *= 0.65). To exclude the influence of allogeneic hematopoietic cell transplantation, we next assessed outcome with censoring on the date of allogeneic hematopoietic cell transplantation in first remission. In fact, there was no significant survival difference in dependence of cytarabine dosage, neither for RFS (2–year RFS HiDAC vs. IDAC, 52.3 vs. 49.7%, HR = 1.008, *p* = 0.97) nor OS (2–year OS HiDAC vs. IDAC, 86.3 vs. 84.1%, HR = 0.999, *p* = 1.00) (Fig. 2B).

**Fig. 3:**
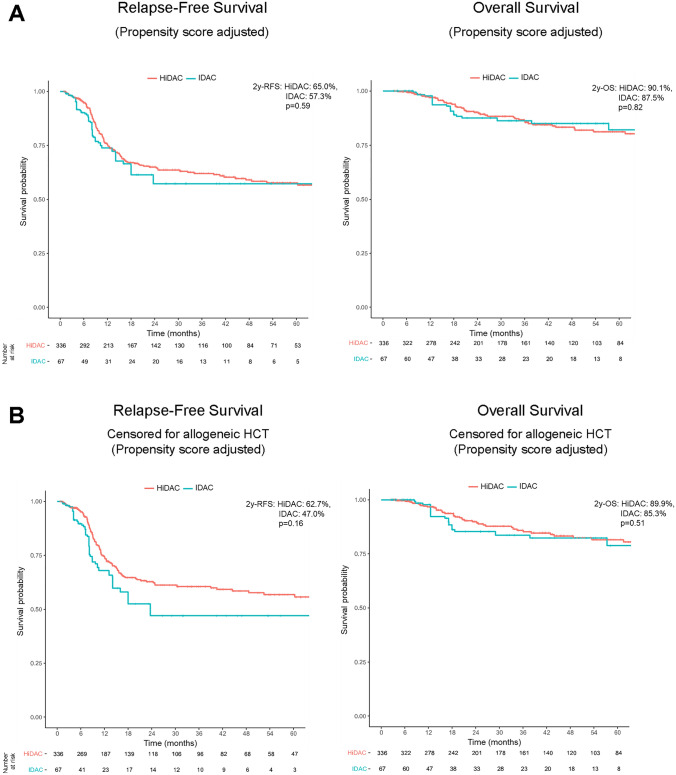
Kaplan–Meier estimates of relapse-free survival (RFS, left) and overall survival (OS, right) for ELN 2017 favorable-risk AML patients after propensity score adjustment (**A**) and censored for allogeneic hematopoietic cell transplantation (HCT) in first complete remission (**B**)

**Fig. 4:**
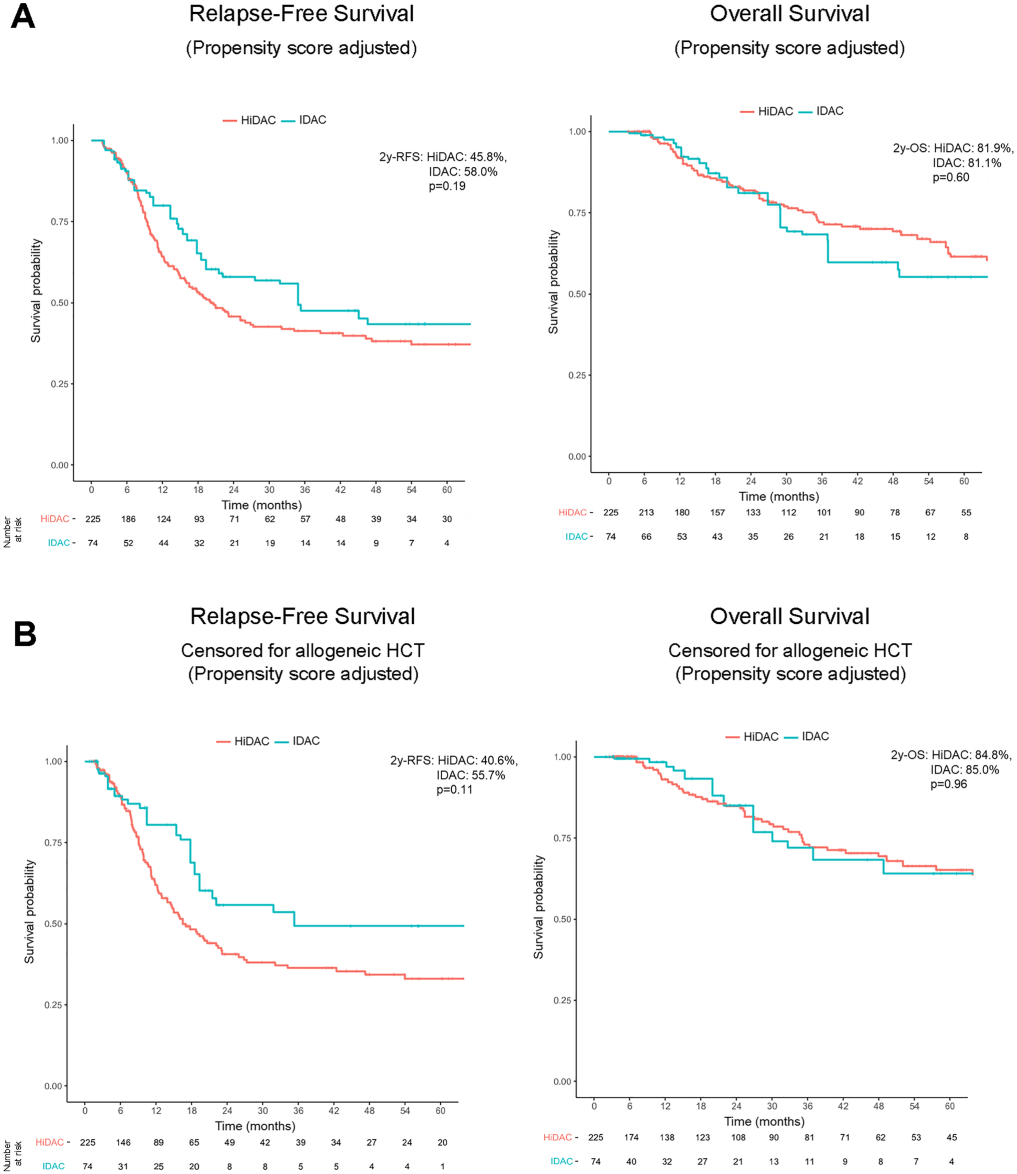
Kaplan–Meier estimates of relapse-free survival (RFS, left) and overall survival (OS, right) for ELN 2017 intermediate risk AML patients after propensity score adjustment (**A**) and censored for allogeneic hematopoietic cell transplantation (HCT) in first complete remission (**B**)

Looking at the subgroup of patients with favorable genetic features according to the ELN 2017 classification, which comprised 336 patients treated with HiDAC and 67 with IDAC, no significance difference for RFS (2-year RFS HiDAC vs. IDAC, 65.0 vs. 57.3%, HR = 1.151, *p* = 0.59) and OS was found (2-year OS HiDAC vs. IDAC, 90.1 vs. 87.5%, HR = 1.092, *p* = 0.82) (Fig. 3A). Though there was no significant difference in the rate of allogeneic hematopoietic cell transplantation in this subgroup, for better comparison, we also censored the ELN favorable cohort on the date of transplantation in first remission. A trend for superior RFS for HiDAC-treated patients appeared with a 2-year survival rate of 62.7% compared to 47.0% for patients consolidated with IDAC, which, however, did not reach statistical significance (HR = 1.453, *p* = 0.16) (Fig. 3B). For OS, there was no significant difference in dependence of cytarabine dose (2–year OS HiDAC vs. IDAC, 89.9 vs. 85.3%, HR = 1.326, *p* = 0.51) (Fig. 3B). Given previously reported evidence suggesting that HiDAC for consolidation is beneficial for core binding factor leukemia (Bloomfield et al. 1998; Miyawaki et al. 2011), we specifically assessed the subgroup of core binding factor AML. With only 20 patients in the IDAC and 116 patients in the HiDAC group, there was no significant difference for RFS and OS (2–year RFS HiDAC vs. IDAC, 59.5 vs. 38.2%, HR 1.742, *p* = 0.16; 2-year OS HiDAC vs. IDAC, 86.6 vs. 97%, HR 1.101, *p* = 0.86), though the small patient number prohibit any conclusion for this subgroup (data not shown). For AML patients at intermediate risk according to ELN 2017 classification, again, there was no significant difference in terms of RFS and OS in dependence of cytarabine dose (Fig. 4A). Given the significant differences in the number of allogeneic-transplanted patients between both cohorts, this subgroup was also censored on the date of allogeneic hematopoietic cell transplantation in first remission. There was a trend for inferior probability of RFS for ELN intermediate risk patients treated with HiDAC compared to IDAC, which, however, was not statistically significant (2-year RFS HiDAC vs. IDAC, 40.6 vs. 55.7%, HR = 0.626, *p* = 0.11) (Fig. 4B). Still, there was no difference in OS (2-year OS HiDAC vs. IDAC, 84.8 vs. 85.0%, HR = 0.979, *p* = 0.96) (Fig. 4B). For ELN adverse risk AML patients, there were no significant differences in outcome in dependence of cytarabine dose, though the numbers of patients were in both groups expectedly low (Fig. S1).

Finally, in multivariable analysis accounting for the influence of ELN risk, number of induction cycles, age, sex, performance and comorbidities, as well as AML type, the dose of cytarabine in post-remission therapy remained not prognostically significant for outcome (Table 2).

**Table 2:** Hazard ratio for relapse-free and overall survival according to multivariable Cox regression models

	Relapse-free survival			Overall survival		
	Hazard ratio	95% CI	*p*	Hazard ratio	95% CI	*p*
Cytarabine dose						
HiDAC	Reference			Reference		
IDAC	0.900	0.650–1.246	*0.530*	1.094	0.721–1.660	*0.670*
ELN risk group						
Intermediate	Reference			Reference		
Favorable	0.704	0.514–0.965	*0.029*	0.435	0.273–0.694	< *0.001*
Adverse	1.147	0.745–1.767	*0.540*	1.771	0.988–3.174	*0.055*
Number of 7 + 3 induction cycles						
1 cycle	1.603	1.195–2.151	*0.002*	1.758	1.221–2.532	*0.002*
2 cycles	Reference			Reference		
Age (per 10 years)	1.012	0.999–1.025	*0.120*	1.021	1.001—1.041	*0.036*
Sex, Male	1.151	0.860–1.539	*0.350*	1.163	0.788–1.718	*0.450*
ECOG, > 1	1.071	0.704–1.629	*0.750*	1.653	1.001–2.728	*0.050*
HCT-CI, > 1	1.220	0.887–1.677	*0.220*	1.425	0.968–2.099	*0.072*
AML type						
de novo	Reference			Reference		
sAML	0.807	0.462–1.410	*0.450*	1.033	0.548–1.947	*0.920*
tAML	1.266	0.795–2.015	*0.320*	1.215	0.657–2.246	*0.540*

*HiDAC/IDAC* high-dose/intermediate-dose cytarabine; *sAML* secondary AML; *tAML* treatment-related AML; *ECOG* clinical performance status according to ECOG criteria; *HCT-CI* hematopoietic cell transplantation-comorbidity index; *7 + 3* induction treatment with standard-dose cytarabine for 7 d and daunorubicin for 3 d; *CR1* first complete remission

### Association of infectious complications with cytarabine dose

Significantly more patients treated with HiDAC suffered from ≥ 3 CTCAE infectious complications (56.7 [95%-CI 52.8–60.6%] vs. 44.1% [95%-CI 36.6–51.7%], *p* = 0.004) (Table 3), which was more striking in patients above 50 years of age (data not shown). The rate of other ≥ 3 CTCAE non-hematological toxicities and secondary malignancies was comparable in both treatment groups (Table 3).

**Table 3:** Non-hematological grade 3 and 4 toxicities according to the Common Toxicity Criteria (CTC)

	IDAC (*n* = 178) (no./no. available (%))	HiDAC (*n* = 642) (no./no. available (%))	*p*
Hemorrhage CTC ≥ 3°	4/177 (2.3)	21/642 (3.3)	*0.656*
Infections CTC ≥ 3°	78/177 (44.1)	364/642 (56.7)	*0.004*
ALAT/ASAT CTC ≥ 3°,	6/177 (3.4)	17/642 (2.6)	*0.786*
Bilirubin CTC ≥ 3°	3/177 (1.7)	10/642 (1.6)	*1.000*
Cardiac CTC ≥ 3°	5/177 (2.8)	19/642 (3)	*1.000*
Creatinine CTC ≥ 3°	3/178 (1.7)	3/642 (0.5)	*0.234*
Other AE CTC ≥ 3°	33/177 (18.6)	137/642 (21.3)	*0.498*
Secondary malignancies	8/177 (5.0%)	21/642 (3%)	*0.571*

*HiDAC/IDAC* high-dose/intermediate-dose cytarabine; *CTC* common toxicity criteria; *ALAT* alanine aminotransferase; *ASAT* aspartate aminotransferase; *AE* adverse event

## Discussion

Since the results from the Cancer and Leukemia Group B (CALBG) study group, which demonstrated an advantage for 3 g/m^2^ cytarabine compared to conventional cytarabine doses of 100 or 400 mg/m^2^ for patients below 60 years of age, the mainstay of conventional consolidation usually has comprised high-dose cytarabine (Mayer et al. 1994). Nevertheless, it is still controversial whether single doses as high as 3 g/m^2^ are necessary. Growing evidence suggests that 1–1.5 g/m^2^ may be similarly efficacious in preventing relapse while being less toxic. For remission induction therapy, Löwenberg et al*.* have clearly shown no advantage of increasing cytarabine above conventional doses while sparing excessive toxicities (Lowenberg et al. 2011). To contribute more information on the ongoing debate on the optimal cytarabine dose level and to add real-world evidence including patients outside clinical trials, we performed this large registry-based study. In this retrospective analysis, we did not detect any significant benefit on outcome after high-dose cytarabine compared to intermediate dosages. Alongside, we observed significantly more infectious complications among HiDAC-treated patients, while there were no other significant differences in tolerability, in particular no increase in early mortality. Our results are in line with findings of a recent retrospective study and a meta-analysis integrating ten randomized clinical trials comparing intermediate and higher cytarabine doses, amongst them eight studies in younger patients. Similar to our findings, high doses of cytarabine were not associated with significant differences in RFS or OS in younger AML patients (Magina et al. 2017; Tangchitpianvit et al. 2021). In addition, a combination of cytarabine with other classic cytotoxic agents did not lead to improved survival (Magina et al. 2017).

Implications that HiDAC for consolidation is beneficial for certain genetic subgroups, in particular core binding factor leukemia (Bloomfield et al. 1998; Kolla et al. 2021; Miyawaki et al. 2011) or *RAS*-mutated AML (Neubauer et al. 2008) compared to conventional cytarabine doses, did not withstand when comparing HiDAC with intermediate doses in different cytogenetic or molecular subgroups (Schaich et al. 2011). We did not observe any significant benefit for ELN favorable-risk AML patients, though there was a non-significant trend for superior RFS among HiDAC-treated patients, which did not result in any difference in OS. Again, this is exactly in line with meta-data from randomized trials (Magina et al. 2017), where prolonged RFS after consolidating HiDAC did not translate into an OS benefit, suggesting a good salvageability of favorable-risk patients in case of relapse. Of note, the risk classification in this meta-analysis was mainly based on cytogenetic criteria compared to the genetic definition by ELN2017 used in our study. Thus, we are able to show conclusive effects of cytarabine doses on survival, indicating that real-world data from a large cohort mirror the results of selected patients participating in randomized trials.

Being based on registry data, our study lacks information on minimal residual disease levels after induction therapy, which could have given valuable insights if intensified consolidation may be beneficial in dependence of residual disease burden. At least, there was no hint that the number of induction cycles among patients who received 7 + 3 induction therapy influenced the outcome in dependence of dose of cytarabine consolidation, as assessed by interaction analyses in multivariable Cox-Model testing (data not shown) and the obvious fact that the significantly higher number of induction cycles among HiDAC-treated patients did not result in survival benefit. The retrospective nature of this study, the risk of not accounting for unknown prognostic relevant factors, which have not been balanced for, as well as the long interval since 2005 might present limitations of this study.

## Conclusions

In summary, this retrospective analysis shows no significant benefit of high-dose cytarabine compared to intermediate dosages in consolidation for AML patients under 65 years of age, independent of ELN risk group. Our results contribute to the growing body of evidence indicating similar efficacy of IDAC and HiDAC consolidation with slightly better tolerability. While evidence on the implications on CBF AML is limited, these real-word data underpin a recommendation for the use of IDAC rather than HiDAC in consolidation chemotherapy.

## Supplementary Information

Below is the link to the electronic supplementary material.

Supplementary file1 (PDF 256 KB)

## Data Availability

All data generated or analyzed during this study are included in this published article and its supplementary information files.
