# Supplementary material for: Intensified cytarabine dose during consolidation in adult AML patients under 65 years is not associated with survival benefit: real-world data from the German SAL-AML registry
Source: J Cancer Res Clin Oncol. 2022 Sep 28;149(8):4611–21. doi: 10.1007/s00432-022-04356-9 (PMC10349710; doi:10.1007/s00432-022-04356-9)
Supplement: Supplementary file 1 — Supplementary file1 (PDF 256 KB) [file 432_2022_4356_MOESM1_ESM.pdf]

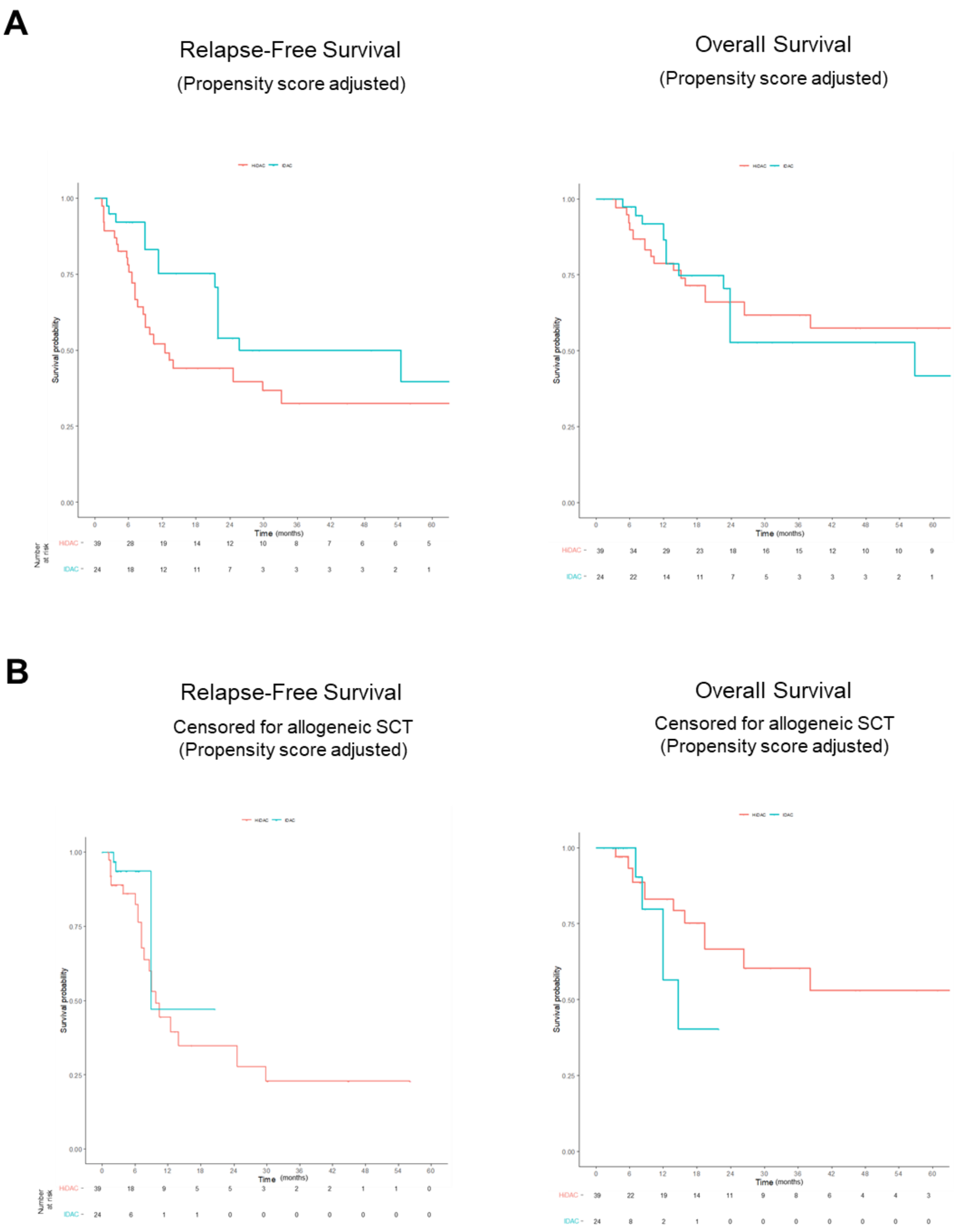

Figure S1. Kaplan-Meier estimates of relapse-free survival (RFS, left) and overall survival (OS, right) for ELN 2017 adverse risk AML patients after propensity score adjustment (A) and censored for allogeneic stem cell transplantation (SCT) in first complete remission (B).
